# Knowledge and exercise of human rights, and barriers and facilitators to claiming rights: a cross-sectional study of female sex workers and high-risk men who have sex with men in Andhra Pradesh, India

**DOI:** 10.1186/s12914-016-0102-2

**Published:** 2016-11-17

**Authors:** Deepika Ganju, Sangram Kishor Patel, Parimi Prabhakar, Rajatashurva Adhikary

**Affiliations:** 1 HIV and AIDS Program, Population Council, 142 Golf Links, New Delhi, 110003 India; 2 India HIV/AIDS Alliance, Sarovar Centre, Secretariat Road, Hyderabad, 500063 India

**Keywords:** Human rights, India, Female sex workers, Men who have sex with men, HIV, Community mobilization

## Abstract

**Background:**

HIV prevention interventions recognize the need to protect the rights of key populations and support them to claim their rights as a vulnerability reduction strategy. This study explores knowledge of human rights, and barriers and facilitators to claiming rights, among female sex workers (FSWs) and high-risk men who have sex with men (HR-MSM) who are beneficiaries of a community mobilization intervention in Andhra Pradesh, India.

**Methods:**

Data are drawn from a cross-sectional survey (2014) among 2400 FSWs and 1200 HR-MSM. Human rights awareness was assessed by asking respondents if they had heard of human rights (yes/no); those reporting awareness of rights were asked to spontaneously name specific rights from the following five pre-defined categories: right to health; dignity/equality; education; property; and freedom from discrimination. Respondents were classified into two groups: more knowledgeable (could identify two or more rights) and less knowledgeable (could identify one or no right). Univariate and bivariate analyses and chi-square tests were used. Data were analyzed using STATA 11.2.

**Results:**

Overall 17% FSWs and 8% HR-MSM were not aware of their rights. Among those aware, 62% and 31% respectively were aware of just one or no right (less knowledgeable); only around half (54% vs 57%) were aware of health rights, and fewer (20% vs 16%) aware of their right to freedom from discrimination. Notably, 27% and 17% respectively had not exercised their rights. Barriers to claiming rights among FSWs and HR-MSM were neighbors (35% vs 37%), lack of knowledge (15% vs 14%), stigma (13% vs 22%) and spouse (19% FSWs). Community organizations (COs) were by far the leading facilitator in claiming rights (57% vs 72%).

**Conclusions:**

The study findings show that awareness of human rights is limited among FSWs and HR-MSM, and a large proportion have not claimed their rights, elevating their HIV vulnerability. For a sustained HIV response, community mobilization efforts must focus on building key populations’ awareness of rights, and addressing the multiple barriers to claiming rights, with a view to creating a safe environment where vulnerable groups can demand and use services without fear of stigma, discrimination and violation of rights.

## Background

Key populations, such as female sex workers (FSWs) and men who have sex with men (MSM), face a disproportionate burden of HIV. Studies from India document that both FSWs and MSM experience widespread human rights violations, including sexual and physical violence [1–5]; unlawful arrest and detention [6]; and HIV-related stigma and discrimination [7–14]; and are deprived of social benefits and entitlements otherwise accessed by the general population [15–17]. These human rights violations are pervasive, undermining HIV prevention efforts and  directly or indirectly elevating HIV vulnerability [18–20]. Forced unprotected sex results in injuries that increase HIV transmission [1, 4, 21]; ﻿a recent study indicates that elimination of sexual violence alone could avert 17–20% of HIV infections among sex workers and clients over the next decade in settings such as Kenya and Canada [20]. Lack of access to banking services increases FSWs’ vulnerability to theft as well as to debt to informal providers (such as money lenders, madams and pimps), reducing their ability to negotiate sexual exchange [22], and stigma and discrimination in health care settings are documented barriers to accessing HIV testing services among key populations [11, 23, 24].

Protecting and promoting the human rights of vulnerable groups, and empowering them to claim their rights, are therefore essential to prevent the spread of HIV. The health and human rights framework has guided the HIV response globally [19]; HIV-related rights, including the right to life, health, privacy and non-discrimination, are enshrined in core human rights conventions, and endorsed by United Nations member states [25–28]. The Indian Constitution makes it mandatory to respect, protect, fulfill, and guarantee human rights without discrimination [29, 30], and the National AIDS Control Organization (NACO) envisions an India where every person living with HIV has access to quality care and is treated with dignity (http://naco.gov.in/about-us/vision-and-value Accessed 24 October 2016). The links between socio-economic and political factors and HIV vulnerability are well documented [31, 32], and therefore, where individuals and communities are able to realize their social, economic, civil and political rights -- to education, free association, information and, most importantly, non-discrimination – the individual and societal impact of HIV are reduced.

HIV prevention interventions in India recognize the need to build a rights-based response for sustaining HIV prevention efforts. In this context, Avahan, the India AIDS initiative of the Bill and Melinda Gates Foundation, initiated a community mobilization program among key populations in 2007 to address structural barriers, including violence, stigma and discrimination within a rights-based framework, in the state of Andhra Pradesh, India [33]. A key program strategy has been to empower key populations-- FSWs and high-risk MSM (HR-MSM)-- to know their social, economic, civil and political rights in the context of the HIV epidemic, and to support them to claim their rights. The Avahan program has mobilized FSWs and HR-MSM populations to build local peer-based networks and community organizations (COs) to work collectively to address barriers to HIV prevention. The Avahan model draws on lessons from the Sonagachi project in West Bengal, which mobilized and empowered FSWs to collectively challenge the factors contributing to vulnerability, leading to the adoption of risk reduction behaviors, the development of social networks and economic empowerment [17, 34].

While evidence suggests that both public health and human rights concerns need to be addressed for a sustained HIV response, few studies in India to date have explored the extent to which prevention programs for key populations have built awareness of human rights and promoted the exercise of rights as a means for vulnerability reduction. To fill this knowledge gap, this study assesses knowledge of human rights, and barriers and facilitators to claiming rights among vulnerable key populations--FSWs and HR-MSM--in Andhra Pradesh, a southern India state. It also explores FSWs’ and HR-MSMs’ collective efficacy—i.e., belief in the power of the community to work together to claim their rights.

## Methods

### Study design

The study is located in Andhra Pradesh, ﻿﻿India,﻿ a state with high HIV prevalence among FSWs (6.3%) and MSM (10.1%) [35]. Our study focuses only on FSWs and HR-MSM and not other key populations, such as injecting drug users (IDUs), because the HIV epidemic in Andhra Pradesh state is largely FSW and MSM driven, while IDU-driven epidemics are concentrated in other states of India such as Manipur and Nagaland [36]. The study draws on data from the Behavioral Tracking Survey (BTS), a cross-sectional survey conducted in 2014 among FSWs and HR-MSM in Andhra Pradesh. The survey aimed to monitor key components of Avahan’s HIV prevention activities, such as condom use, and to assess knowledge of specific human rights, exercise of rights, and barriers to and facilitators in claiming rights.

### Sampling

FSWs and HR-MSM were recruited from six program districts (Ananthapur, Chittor, Karimnagar, Khammam, Nalgonda and Warangal) for the survey. These districts were purposively selected based on their geographical location and socio-cultural variability. For both groups (FSWs and HR-MSM), a sample size of 400 completed interviews was calculated for each district, based on prevalence of consistent condom use. The sampling frame was prepared by taking the number of FSWs/HR-MSM registered in each CO. A two-stage random cluster sampling method was used to select respondents. In the first stage, COs within different clusters/wards were selected using the probability proportional to size procedure. In the second stage, the required number of FSWs/HR-MSM was randomly selected for interview within each selected CO. A total sample of 2400 FSWs and 1200 HR-MSM was collected.

### Data collection

To be eligible for participation in the FSW survey, individuals had to be female, aged 18 years or more, and have had sex in exchange for cash/kind in the one month prior to the survey. Eligibility for participation in the MSM survey included being male, aged 18 years or older, identified to have had sex with another male in exchange for cash/kind in the past one month, and cruising from one place to another, soliciting clients or hanging out at any suitable place including street corners, highways and pick-up points within the operational area.

Interviews were conducted by trained investigators with verbal and written skills in Telugu, the local language of Andhra Pradesh. The survey instrument was developed in English and translated into Telugu. The interview schedule was pre-tested in communities similar to the survey sites. All the interviews were held in a private location specifically hired for the survey, or in a location convenient to the study participants. Each interview lasted approximately 30–45 min. Field staff checked the data immediately after the interviews to ensure accuracy and completeness of the questionnaires. A user-written computer program in CSPro (version 5.0) was used for double data entry by trained data entry staff.

### Ethics statement

The original BTS design and questionnaires were approved by the institutional review boards of Family Health International and the Karnataka Health Promotion Trust. Verbal consent was obtained from all respondents prior to participation in the interview. No names and addresses were recorded on the questionnaires. Participants could opt out of the survey at any time. Participants were not compensated for their time in the survey but were referred to project services run by implementing agencies in the study districts.

### Measures

#### Socio-demographic and behavioral variables

The socio-demographic and behavioral variables included were age (<30 years, ≥ 30 years); formal education (no, yes); marital status (never married, currently married, and widowed/divorced/separated/deserted); usual place of practicing sex work (rural, urban/ semi-urban); and current living status (living with spouse/family members, living with others and living alone).

#### Human rights

*Knowledge of human rights*: In this study, awareness of human rights was assessed based on a single item question on whether the respondent had heard of human rights (yes/no); those reporting "yes" were asked to name specific rights. Spontaneous multiple responses were recorded into each of the five following pre-defined categories: right to access health services; right to dignity and equality; right to education; right to property; right to be free from stigma and discrimination. Based on this, respondents were classified into two groups: those who could identify two or more of the five rights were considered to be more knowledgeable about rights and those who could identify one or none of the specific rights were considered to be less knowledgeable.

*Claiming human rights, and barriers and facilitators to claiming rights:* Respondents who were aware of rights were asked if they had ever attempted to claim their rights (yes/no); those who answered "yes" were asked to name the organization/staff that had assisted them in claiming these rights; spontaneous multiple responses were recorded into each of the seven following pre-defined categories --COs, health clinics (including anti-retroviral treatment [ART] clinics and designated sexually transmitted infection [STI]/reproductive tract infection [RTI] clinics), the District Collector, legal/para-legal staff (including District Legal Service Authorities [DLSA] and para-legal volunteers), District AIDS Prevention Control Unit (DAPCU) staff, the police and others. To assess barriers to claiming rights, respondents who were aware of rights were asked to name the barriers they perceived to claiming rights. Spontaneous multiple responses were recorded into each of the seven following pre-defined categories: neighbors, regular partner/spouse, lack of awareness, stigma, government officials, judiciary and others.

#### Collective efficacy

In this study, collective efficacy was assessed based on the following measure-- FSWs were asked to respond to a direct question: "How confident are you that sex workers can organize to speak for their rights” and HR-MSM were asked “How confident do you feel that MSM can organize and fight against Section 377” of the Indian Penal Code (Section 377 criminalizes sexual activities “against the order of nature,” including same-sex relations). Those indicating they were not confident or somewhat confident were coded as 1 (not confident), and those reporting very confident or completely confident were coded as 2 (very confident).

### Statistical analysis

Bivariate and descriptive analyses (i.e., standard deviations, and proportions) were undertaken for all socio-demographic and human rights variables. Respective *p*-values were calculated using the chi-square test. All analyses were conducted using STATA (version 11.2).

## Results

### Profile of respondents

Significant differences were observed in some socio-demographic characteristics of FSWs and HR-MSM (Table 1). While the majority (57%) of FSWs were in the older age group (≥30 years), did not have any formal education (56%) and were currently married (67%), in contrast, over three-fifths (62%) of HR-MSM were in the younger age group (<30 years), 86% had a formal education and three-fifths were never married. The majority of FSWs and HR-MSM (77% vs 71%) were living with their spouse or family members; while 11% of FSWs were either living alone or living with others, one-fifth of HR-MSM were living on their own. No significant differences were observed with regard to usual place of practicing sex work across the two groups, with just over half of both FSWs and HR-MSM reporting that they generally practiced sex work in urban areas.

**Table 1:** Profile of female sex workers and high-risk men who have sex with men in Andhra Pradesh, India, 2014

Background characteristics	FSWs (*N* = 2400)	HR-MSM (*N* = 1200)	*P* value^1^
Age			
< 30 years	43.3 (1040)	62.3 (748)	<0.0001
≥ 30 years	56.7 (1360)	37.7 (452)	<0.0001
Mean (SD)	30.9 (5.8)	28.7 (5.8)	
Education			
No formal education	56.3 (1350)	13.8 (165)	<0.0001
Some formal education	43.8 (1050)	86.3 (1035)	<0.0001
Marital status			
Never married	5.0 (119)	60.3 (723)	<0.0001
Currently married	66.5 (1596)	32.5 (390)	<0.0001
Widowed/deserted/separated/ divorced	28.5 (685)	7.3 (87)	<0.0001
Usual place of practicing sex work			
Rural	46.7 (1121)	45.3 (543)	0.4267
Urban/semi-urban	53.3 (1279)	54.7 (657)	0.4267
Current living status			
Living with spouse/ family members	77.2 (1854)	70.8 (849)	<0.0001
Living with others	10.5 (251)	8.1 (97)	<0.0001
Living alone	11.4 (274)	21.2 (254)	<0.0001

*FSWs* female sex workers, *HR-MSM* high-risk men who have sex with men

^1^Chi-square test

### Knowledge of human rights

A significantly larger proportion of HR-MSM than FSWs (93% vs 83%; *p* < 0.0001) reported awareness of human rights (Table 2). Among those aware of rights, notable differences were observed across the groups in terms of more knowledge/less knowledge of rights, with a significantly larger proportion of HR-MSM than FSWs (69% vs 38%; *p* < 0.0001) reporting awareness of two or more rights. Knowledge of specific rights was low: in both groups, among those aware of rights, two-fifths or more could not name any of the five specific rights, and only 20% FSWs and 16% of HR-MSM were aware of their right to be free from stigma and discrimination. Just over half (54% FSWs and 57% HR-MSM) were able to name the right to health. A significantly larger proportion of HR-MSM than FSWs were aware of the right to education (47% vs 40%; *p* < 0.0001) and the right to property (40% vs 27%; *p* < 0.0001). Notably, 83% of HR-MSM were aware of Section 377 (not shown in tabular form).

**Table 2:** Knowledge and exercise of human rights among female sex workers and high-risk men who have sex with men in Andhra Pradesh, India, 2014

Indicators	FSWs	HR-MSM	*P* value^1^
Aware of human rights	*N* = 2400	*N* = 1200	
Yes	83.3 (2000)	92.5 (1110)	<0.0001
No	16.7 (400)	7.5 (90)	<0.0001
Among those aware of human rights:	*N* = 2000	*N* = 1110	
Knowledge of specific rights:^a^			
Right to access health services	54.3 (1086)	57.4 (637)	0.0948
Right to education	39.8 (795)	47.2 (523)	<0.0001
Right to dignity and equality	34.9 (697)	54.8 (608)	0.9572
Right to property	26.6 (532)	40.4 (448)	<0.0001
Right to freedom from stigma and discrimination	20.4 (408)	15.8 (175)	0.0012
Knowledge of rights^b^			
Less knowledgeable	62.0 (1488)	30.7 (369)	<0.0001
More knowledgeable	38.0 (912)	69.3 (831)	<0.0001
Able to claim rights			
Yes	73.2 (1463)	82.9 (921)	<0.0001
No	26.8 (537)	17.1 (190)	<0.0001
Collective efficacy	*N* = 2000	*N* = 1001	
Confident that sex workers can organize to speak for their rights			
Not confident	35.8 (716)	NA	
Very confident	64.2 (1284)	NA	
Confident that MSM can organize and fight against Section 377^c^			
Not confident	NA	56.0 (561)	
Very confident	NA	44.0 (440)	

*FSWs* female sex workers, *HR-MSM* high-risk men who have sex with men

^1^Chi-square test. *NA* not asked

^a^Multiple responses

^b^Less knowledgeable = aware of one or no human rights; More knowledgeable = aware of 2 or more human rights

^c^Among those aware of Section 377

### Claiming human rights

Among those aware of rights, significantly more HR-MSM than FSWs (83% vs 73%; *p* < 0.0001) reported being able to claim their rights. In terms of collective efficacy, over one-third of FSWs (36%) were not confident that sex workers could organize to speak for their rights, and among HR-MSM aware of Section 377, more than half (56%) reported that they were not confident that MSM can organize and fight against Section 377.

### Facilitators and barriers in claiming human rights

**Fig. 1:**
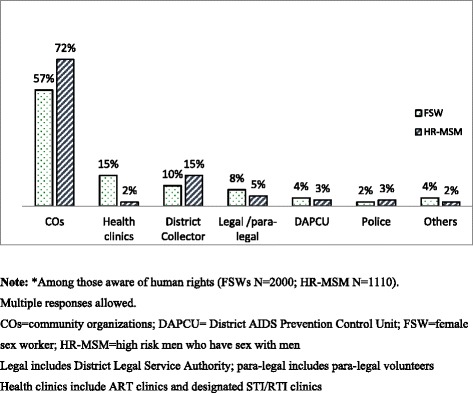
Facilitators in claiming rights as reported by female sex workers and high-risk men who have sex with men in Andhra Pradesh, India, 2014*

**Fig. 2:**
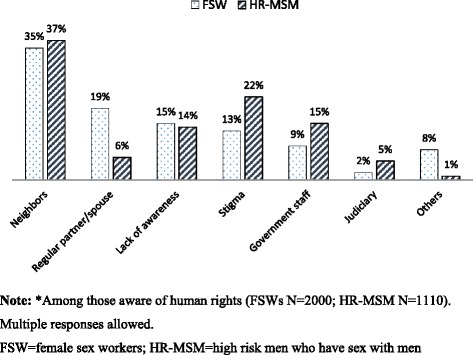
Barriers to claiming rights as reported by female sex workers and high-risk men who have sex with men in Andhra Pradesh, India, 2014*

Most respondents in both groups reported that COs were the key facilitator in claiming rights (Fig. 1). Among FSWs aware of rights, over half (57%) had claimed their rights through COs, while fewer (10-15%) reported that health clinics and the District Collector had facilitated exercise of rights, and 8% had claimed their rights through legal/para-legal authorities. Among HR-MSM aware of rights, almost three-quarters (72%) had been facilitated by COs to claim their rights; far fewer reported exercise facilitated by the District Collector (15%) and legal and para-legal staff (5%). Over one-third of respondents in both groups (35% of FSWs vs 37% of HR-MSM) perceived neighbors to be the main barrier to claiming rights (Fig. 2); other barriers for FSWs were regular partners and husbands (19%), lack of awareness (15%) and stigma (13%), while for HR-MSM, stigma (22%), government staff (15%) and lack of awareness (14%) were reported.

## Discussion

While there is growing recognition of the need to protect the rights of vulnerable populations for a sustained HIV response, and although the health, social, economic, civil and political rights of all persons are guaranteed under international frameworks and national laws, our study shows that not all key populations, particularly FSWs, are aware of their rights in Andhra Pradesh, India. As a result, these marginalized groups may not appreciate the extent of rights violations, undermining their ability to seek redress, legal advice or support. Further, not all these sub-groups have claimed their rights, depriving them of control over their working conditions, ability to consistently engage in safer sexual behaviors, seek non-discriminatory health services and access social and economic entitlements and schemes, thereby directly or indirectly elevating HIV vulnerability These findings are of concern given that marginalized groups are at elevated risk of experiencing human rights violations [19, 20]. Building awareness and protecting the rights of marginalized groups would help to reduce HIV vulnerability and meet the Sustainable Development Goals (SDGs) 3,5 and 8, pertaining to good health and wellbeing, gender equality and women’s empowerment, and the promotion of full and productive employment for all.

Our study examined awareness of five rights considered central to a rights-based approach, which are being addressed through the ongoing HIV prevention program in the state [33]. Notably, knowledge of these rights was far from universal in both groups, but more so among FSWs; underscoring these findings, lack of awareness was also an identified obstacle to claiming rights in our study. An unexpected finding was that both FSWs and HR-MSM were least aware of the right to be free from stigma and discrimination (reported by one-fifth or less), although both groups face widespread discrimination and marginalization in multiple spheres [7–9, 19, 37]. Further, just 35% of FSWs and 55% of HR-MSM were aware of the right to dignity and equality. Moreover, although marginalized communities face rampant human rights abuses in health settings, ranging from inadequate or inappropriate services to denial of care, to discriminatory treatment [8, 11, 19], and all the respondents are beneficiaries of an upscaled HIV prevention program with a focus on health, awareness of the right to health was far from universal. Limited awareness of these critical rights, as well as other related rights– right to property and right to education–could compromise key populations’ exercise of rights and claiming entitlements, thereby creating an environment for HIV transmission. Efforts to build knowledge of human rights must be strengthened if key populations are to demand and claim their rights, and improve their health and well-being [11].

Our findings that FSWs are significantly disadvantaged in terms of knowledge of human rights as well as claiming their rights have wider implications in the Indian context where unequal gender relations, pervasive poverty and social marginalization are associated with sexual violence, economic insecurity, housing discrimination, as well as risky sex and HIV vulnerability among FSWs [16, 38]. Protecting the rights of FSWs, including their sexual and reproductive rights; addressing sexual and physical violence perpetrated by partners, pimps, and the police; promoting equal access to education and work opportunities; and addressing gender barriers in claiming social and economic entitlements and health services, and discrimination in inheritance are important in reducing the impact of the HIV epidemic on this vulnerable group.

Effective HIV prevention depends on the ability of key populations to claim their rights and access services. Notably, legal and rights literacy and access to justice, remedies and redress are among the key human rights programs that UNAIDS recommends in the framework of the HIV response [39]. Although the primary objective of the HIV prevention program in Andhra Pradesh is to strengthen the capacity of key populations to understand and exercise their rights, however, as shown in this study, a considerable proportion of FSWs and HR-MSM had never claimed their rights. Prior studies have shown that abuse is perpetrated with impunity due to the recognition that key populations face barriers in seeking justice [19], and rights violations are rarely reported due to a sense of futility that perpetrators will be punished, and fears of further violence [40].

Our study has identified several obstacles to claiming rights. Most often community members--including neighbors, and in the case of FSWs regular partners/spouse--were barriers to claiming rights. These findings corroborate earlier research; a study in India documents that fear of rejection by community members/friends is associated with MSMs’ limited ability to adopt safer sex behaviors and utilize health services [8]. Similarly, FSWs are unable to exercise their rights in violent relationships with regular partners, as violence compromises their ability to negotiate condom use, particularly if violence is sustained over time [1, 2, 41]. Stigma was identified as a major obstacle to claiming rights, particularly among MSM [7–9, 42, 43]; as reported, marginalized populations face overlapping and multiple stigma (e.g., related to homophobia, sex work, sexual orientation and poverty), enacted within unequal power structures, elevating vulnerability to human right abuses and compromising their ability to challenge abuse or claim their rights, including to necessary prevention and treatment services [44]. District-level government officials can assist key populations to generate identity documents and apply for social entitlements and financial schemes, and the judiciary can provide legal information and redress in cases of rights violations; however, findings show that both were reported to be barriers to claiming rights.

Among both FSWs and HR-MSM, the leading facilitator in claiming rights were local COs; this finding is not surprising as all respondents are members of COs, and given evidence that sex worker-led, rights-based programs such as Avahan and Sonagachi, are effective in promoting rights across the health, social and law enforcement sectors, including linking sex workers to bank accounts, social inclusion schemes and health insurance, reducing violence, and empowering FSWs to challenge social exclusion and threats to their dignity [1, 45–47]. Notably, in our study, ﻿jus﻿t 15% ﻿﻿or fewer respondents﻿ identified the District Collector (who is the local administrator) and even less (<5%) described the DAPCU as facilitators, although DAPCUs are mandated to empower marginalized groups to access social benefits and protection schemes [48]. As expected, the police were not perceived as facilitators in claiming rights; the police are widely documented to perpetrate rights abuses among FSWs and HR-MSM [19, 49], particularly in the context of the quasi-criminalization of sex work and the criminalization of homosexual relations in India, and seldom take action when sex workers report violence [1, 8, 19, 50]. Further, although health providers play a central role in providing information and services for HIV prevention and care, health staff were not the leading facilitator in claiming rights.

Our findings provide evidence that community mobilization interventions must go beyond the provision of individual level prevention interventions, and prioritize efforts to build awareness among FSWs and HR-MSM of their rights in the context of the HIV epidemic, and empower them to demand non-discriminatory services so that they can make choices and adopt safer behaviors [38]. In India, community-led interventions among marginalized groups have resulted in measurable improvements in sex workers’ quality of life, self-confidence, and agency, and social and economic outcomes, including increased social capital [19, 20, 23, 51].

Interventions are needed at multiple levels. Key populations must be provided legal education and legal aid services to access justice. Creating a “Know Your Rights” resource for sex workers indicating rights applicable upon arrest or detention, in addition to other rights and remedies would be useful. Information materials must be designed keeping in mind the literacy levels of the intended audience, and disseminated through multiple channels including the media (social media, TV, radio, print, and internet), peers, and telephone helplines. Further, key populations must be supported to report violence by different perpetrators, even if the perpetrator is an intimate partner; as documented, despite ongoing HIV prevention programs, FSWs are reluctant to report partner violence due to the perception that the degree of violence is not severe and lack of awareness of their legal rights [1, 2, 41, 52].

Further, community mobilization programs must address stigma by building awareness among community members (including the family, peers and sexual partners) regarding the inviolable rights of key populations, including those involved in sex work and those engaging in homosexual sex, and promote the integration of these marginalized groups into society by encouraging their participation at public meetings. Community-level meetings could provide a forum for discussions and awareness building on stigma and the promotion of key populations’ rights. Community empowerment approaches are cost-effective [53], and have demonstrated reduced stigma and related outcomes, with members of COs (including community advocacy groups and crisis response groups) in India reporting reduced perceived discrimination, violence and police harassment, as well as economic empowerment and social support [15–17, 22, 53–57].

In the context that government staff and the judiciary were identified as barriers to claiming rights, and only a small proportion of key populations reported that the police, para-legal authorities and the district administration had supported them to claim their rights, efforts are needed to build linkages with key stakeholders--legal authorities government agencies, and the police—to facilitate key populations to access services, as well as to legal redress for persons whose rights have been violated. Peer educators and outreach workers can reach marginalized groups with information and services; existing peer and outreach networks should be utilized to provide FSWs and HR-MSM with information on human rights and laws related to HIV and rights violations, and link them to sources of legal support and services. Training community members as para-legals to provide legal advice, mediation services and education on rights issues, accompany key populations to health clinics and to court, and assist them with bail applications could be effective. Additionally, programs must sensitize the police on HIV related issues, including laws related to the rights of sex workers and MSM, the importance of reaching out to populations at risk and addressing domestic and sexual violence and other rights violations. Further, given our findings that health staff were not key facilitators in accessing rights, and prior evidence of human rights violations in health facilities [9, 58], HIV prevention interventions must build awareness among health providers of key populations’ right to informed consent, confidentiality and treatment, and equality and non-discrimination in accessing services across the continuum of care [19].

While community mobilization interventions must be scaled up for a sustained response, special efforts are needed to build collective efficacy, leadership and advocacy skills among both FSWs and HR-MSM. Although all the respondents have been exposed to community mobilization interventions, a large proportion of both FSWs and HR-MSM reportedly perceived that their group would not be able to advocate and work collectively to claim their rights, whether as sex workers or against laws that criminalize same-sex relations. While community empowerment fosters resilience among sex workers, the finding that a larger proportion of HR-MSM than FSWs were not confident to work collectively to claim their rights suggests that it may be more difficult for key populations to challenge punitive laws (such as Section 377, which criminalizes homosexuality) than to promote sex workers’ rights more generically. Programs must focus on expanding key populations’ exposure to the program and empowering them to collectively challenge the factors contributing to their vulnerability.

This study is one of the first to explore key populations’ vulnerability to HIV in India in the context of knowledge and exercise of rights. Our study goes beyond an understanding of health rights, to explore the larger political, social, civil and economic rights that also impact HIV vulnerability. However, given the limited information available on this issue, further research is needed to understand the extent and nature of human rights violations against marginalized groups in different settings, identify the determinants of human rights violations, assess the effect of human rights violations on HIV and demonstrate that rights-based interventions lead to positive health and related outcomes.

While the study findings have several important programmatic implications, the results must be interpreted in light of certain limitations. For one, information on knowledge and claiming rights are based on self-reports and may therefore be vulnerable to social desirability and reporting biases. However, the use of trained and experienced research staff may have increased respondents’ comfort and reduced reporting bias. Second, the study was conducted among FSWs and HR-MSM who are beneficiaries of a community mobilization program in Andhra Pradesh; this socio-cultural and program context may not be similar to those of key populations residing in other settings in Ind﻿ia, and hence the results may not be generalizable ﻿﻿to﻿﻿ all marginalized groups in India. Nonetheless, these limitations do not compromise the internal validity of the data.

## Conclusions

While rights abuses against key populations are well-documented, this study took a step towards understanding key populations’ knowledge and exercise of human rights, and the barriers and facilitators they face in claiming rights. Findings show that although key populations --FSWs and HR-MSM-- in Andhra Pradesh, India are beneficiaries of an upscaled HIV prevention program with a focus on community mobilization, awareness of human rights, particularly among FSWs, is far from universal. Not all are knowledgeable about specific rights, including the right to health and the right to be free from stigma and discrimination, and a large proportion had not claimed their rights. As a result, these marginalized groups are unable to seek redress, legal advice or support, and lack the ability to adopt safe behaviors and access non-discriminatory services, elevating their HIV vulnerability. The study findings also show that COs are the key facilitator in claiming rights, while the main barriers to claiming rights are the community, stigma, government staff, lack of awareness, and among FSWs their regular partners. These findings have important programmatic implications. For a sustained HIV response, community mobilization efforts must prioritize building key populations’ awareness of rights and empower them to demand and use services without fear of stigma, discrimination and rights violations. Interventions must address the barriers to claiming rights by providing legal education and legal support to key populations to access justice; empower key populations to report human rights abuses, even when perpetrated by intimate partners; address stigma by building community awareness of the inviolable rights of key populations; and build linkages with key stakeholders—the police, health staff, district administrators, community members and legal practitioners-- to create a safe environment where key populations can access services and seek redress for rights violations.

## Abbreviations

ART: Anti-retroviral treatment
BTS: Behavioral Tracking Survey
CO: Community organization
DAPCU: District AIDS Prevention Control Unit
DLSA: District Legal Service Authorities
FSW: Female sex worker
HR-MSM: High-risk men who have sex with men
IDU: Injecting drug user
RTI: Reproductive tract infection
STI: Sexually transmitted infection
